# Childhood intestinal parasitic infection and sanitation predictors in rural Dembiya, northwest Ethiopia

**DOI:** 10.1186/s12199-018-0714-3

**Published:** 2018-06-22

**Authors:** Zemichael Gizaw, Tsegaye Adane, Jember Azanaw, Ayenew Addisu, Daniel Haile

**Affiliations:** 1 Department of Environmental and Occupational Health and Safety, Institute of Public Health, College of Medicine and Health Sciences, University of Gondar, Gondar, Ethiopia; 2 Department of Parasitology, School of Biomedical Science, College of Medicine and Health Sciences, University of Gondar, Gondar, Ethiopia

**Keywords:** Intestinal parasitic infections, WASH predictors, Children aged 6–59 months, Rural Dembiya

## Abstract

**Background:**

Intestinal parasites are a common problem in the world. The greater proportion of infections is associated with poor water, sanitation, and hygiene (WASH). This study was conducted to assess intestinal parasites, WASH condition, and their association in rural Dembiya, northwest Ethiopia.

**Methods:**

A cross-sectional study was employed. Two hundred twenty-five children aged 6–59 months were included. Mothers were interviewed using a structured questionnaire, and the living environment was observed using checklists. Kato-Katz technique was used to determine the intensity of parasitic infections. *Escherichia coli* (*E. coli*) was used as a biological indicator for drinking water quality. Multivariable binary logistic regression analysis was conducted to identify WASH predictors of parasites on the basis of adjusted odds ratio (AOR) with 95% confidence interval (CI) and *p* < 0.05.

**Results:**

The prevalence of intestinal parasites was 25.8% (95% CI = 20.3–32.0%). *Ascaris lumbricoides* (78%), hookworm (12%), *Hymenolepis nana* (7%), *Enterobius vermicularis* (5%), *Schistosoma mansoni* (3%), *Giardia lamblia* (3%), and *Trichuris trichiuria* (2%) were identified infections. Intestinal parasites were associated with poor child hand washing practice [AOR = 3.86, 95% CI = 1.53, 9.75], unprotected water sources [AOR = 7.79, 95% CI = 3.30, 18.40], access to water below 20 l/c/d [AOR = 3.05, 95% CI = 1.28, 7.23], poor food safety[AOR = 4.33, 95% CI = 1.62, 11.58], and poor sanitation [AOR = 5.01, 95% CI = 1.56, 16.16].

**Conclusion:**

*A. lumbricoides*, hookworm, *H. nana*, *E. vermicularis*, *S. mansoni*, *G. lamblia*, and *T. trichiuria* were identified. Child hand washing practice, service level of water supply, water sources, food safety, and sanitation were associated with intestinal parasites. WASH promotion is needed to prevent infections.

## Background

Intestinal parasitic infections are major public health problems in the world, especially in developing countries causing under nutrition, anemia, intestinal obstruction, and mental and physical growth retardation [1]. Intestinal parasitic infections, mainly *Ascariasis*, *Trichiuriasis*, and hookworm, are common clinical disorders in man, with resultant impairments in physical, intellectual, and cognitive development [2]. About 3.5 billion people (the majority of these cases were children) in the world were infected with intestinal parasites caused by helminths and protozoa during 2009 [3], and about 1.45 billion cases were due to soil-transmitted helminth (STH). Out of 1.45 billion infections due to STHs, 819.0 million were infected with *A. lumbricoides*, 464.6 million with *T. trichiura*, and 438.9 million with hookworm [4]. Of the 4.98 million years lived with disability (YLD) attributable to STH, 65% were attributable to hookworm, 22% to *A. lumbricoides*, and the remaining 13% to *T. trichiura* [4].

Intestinal parasitic infections are still major public health problems in sub-Saharan Africa. In sub-Saharan African countries, millions of people are infected with at least one intestinal parasitic infection. A global estimate shows that in the region, 118 million people (13.6% of the population), 118 million people (13.6% of the population), and 101 million people (11.6% of the population) were infected by hookworm, *A. lumbricoides*, and *T. trichiura*, respectively, during 2010 [4] of which the majority were children [5].

In Ethiopia and other low-income countries including those in sub-Saharan Africa, the population suffers from a huge burden of potentially preventable diseases such as intestinal parasitic infections [6]. In Ethiopia and two other sub-Saharan African countries (Nigeria and the Democratic Republic of the Congo), STH infections account 8% of the global STH infections [4]. In the country, intestinal parasitic infections are prevalent among children. The global estimate during 2005 showed that 4882 school-aged children were infected with hookworm, 1956 with *A. lumbricoides*, 1983 with *T. trichiura*, and 7357 with other STH species [7].

Intestinal parasitic infections are caused by different factors, and the greater proportion of infections is associated with poor WASH conditions and most of the infections are fecal-oral [8–15]. WASH promotion is a planned and systematic activity to enable people to take action to improve WASH and to prevent or mitigate WASH-related diseases and to provide a practical way to facilitate community participation and accountability. It includes (i) access to facilities like community water supply system, waste management or sanitation facilities, and household-level technologies and materials; (ii) access to software services or community behavioral change services; and (iii) establish enabling environments like policy improvement, community organizations, sanitation financing, public-private partnership, and institutional strengthening [16]. In cognizant of the role of WASH promotion to prevent the transmission of intestinal parasitic infections, the University of Gondar in collaboration with Neglected Tropical Diseases Advocacy Learning Action (NALA) Foundation has been implementing WASH promotion project in rural Dembiya. This community-based cross-sectional study was conducted as a baseline survey to assess the prevalence and WASH predictors of intestinal parasitic infections among children aged 6–59 months**.**

## Methods

### Study design and description of study settings

A community-based cross-sectional study was conducted in May 2017 in rural Dembiya. Dembiya is one of the woredas in North Gondar Zone, the Amhara National Regional State, Ethiopia. Dembiya is bordered on the south by Lake Tana, on the southwest by Takusa, on the west by Chilga, on the north by Lay Armachiho, and on the east by Gondar Zuria [17]. The district finance and economic development report in June 2017 showed that Dembiya district had a total population of 326,686, of whom 162,477 were men and 164,209 were women with 1:1 sex ratio. Under-five children accounted for 12.22% (39,927) of the total population [18]. Hygiene and sanitation-related communicable diseases were highly prevalent in the area. During June 2017, intestinal parasitic infections and diarrheal diseases were the top four and five prevalent diseases, which accounted 5161 (9.97%) and 4981 (9.62%), respectively. The population in the area had poor access to sanitation. During June 2017, clean water and latrine coverage in the district was 26.60 and 55%, respectively [19].

### Sample size determination, sampling techniques, and sampling procedures

The sample size was determined using single population proportion formula with the following assumptions: *p* = 85.1% (prevalence of intestinal parasitic infections among children aged 6–59 months in Shesha Kebkele, Wondo Genet, Southern Ethiopia during 2010) [20], 95% confidence interval, and a 5% margin of error (*d*).

$$ n=\frac{{\left({z}_{\raisebox{1ex}{$\alpha $}\!\left/ \!\raisebox{-1ex}{$2$}\right.}\right)}^2p\left(1-p\right)}{d^2}=\frac{(1.96)^20.851\left(1-0.851\right)}{0.05^2}=195 $$

By taking 15% nonresponse rate, the final sample size became 225. Therefore, a total of 225 children aged 6–59 months were selected from five rural kebeles. The study subjects were selected by systematic random sampling technique. We spun an arrow at the center of the kebeles to identify the first household. The first household was selected randomly from 22 houses located in the direction of the arrow. The older one was selected in this study for households which had more than one child.

### Data collection procedures

Data were collected using four different data collection methods. Children were provided a plastic stool container and asked to bring approximately 15 g of their own stool. For direct stool examination, a drop or drops of saline were placed on a slide. Approximately 0.05 g of stool specimen was placed using an applicator stick and mixed with a drop of saline and covered by cover slide. Finally, the specimen was examined under the microscope at low power (× 10 objective) and high power (× 40 objective) magnifications for the identification of intestinal parasites [21]. For the Kato-Katz, a small amount (approximately 2 g) of feces was placed on a piece of scrap paper. The stool was pressed on the top of the screen of the fecal specimen using the applicator stick. After the upper surface of the screen is scraped to sieve the fecal specimen, the template was placed on a clean microscopic slide and filled with the sieved fecal specimen. Then the template was removed carefully so that the entire fecal specimen remained on the slide. The remained fecal specimen was covered with glycerol-soaked cellophane strip and examined on the × 10 objective microscope [21]. Stool specimen was analyzed immediately after collection. Examination of parasites was done by certified laboratory technicians. Water samples were taken from individual households at point of use using sterilized sampling bottles, and the samples were transported to the central laboratory within 4 h with cold chain. Moreover, the overall condition of water sampling was recorded on a checklist. The living environment and housing conditions were observed using checklists. Mothers were interviewed using a structured questionnaire. Field supervisors had also monitored the data collection process to assure data or sample quality.

### Measurement of study variables

#### Prevalence of intestinal parasitic infection

This is defined as the presence of one or more intestinal parasite species among children. The intensity of parasitic infections was also determined based on the number of parasitic eggs per gram (epg) of the stool sample. The intensity of *Ascaris lumbricoides* was classified into a light infection (1–4999 epg), moderate (5000–49,999 epg), and heavy (greater than 50,000 epg). The intensity of hookworm was classified into a light infection (1–1999 epg), moderate (2000–3999 epg), and heavy (greater than 4000 epg). The intensity of *Schistosoma mansoni* was also classified into a light infection (1–99 epg), moderate (100–399 epg), and heavy (greater than 400 epg) [22–24]**.**

#### Hygiene of children and mother’s hand washing practice

The general cleanliness of the children was taken as “clean” if no any visible dirt on their clothes their figures and hand have no dirt, their hair is neat, their face is neat and wash their body regularly, and if they wear shoes. Mothers’ or caregivers’ hand washing practice was taken as “good” if they washed their hands with soap before meal, after defecation, after handling baby’s diaper or feces, after meals, before feeding a child, before food preparation, and after handling rubbish or animals.

#### Drinking water quality and access level

Drinking water quality was taken as “good” if zero *E. coli* was found and “not good” if one or more *E. coli* were found in 100 ml water sample [25]. Risk of drinking water was also classified into a different risk category based on colony-forming units (cfu) per 100 ml of water. Risk category was taken as conformity (0 cfu/100 ml), low risk (1–10 cfu/100 ml), intermediate risk (10–100 cfu/100 ml), high risk (100–1000 cfu/100 ml), and very high risk (41,000 cfu/100 ml) [24, 26]. Drinking water sources were taken as “protected” if the community fetched water from protected springs or protected wells or public taps and “unprotected” if the water sources were rivers, unprotected springs, and unprotected wells. Drinking water supply service level was taken as “no access” and “basic access” if households had collected below 20 l per capita per day (l/c/d) and 20 l/c/d and above water, respectively.

#### Food safety practices

Food safety practice was taken as “good” if a person who is responsible for food preparation did not prepare when he or she has diarrhea or other communicable diseases, if households washed fruits or vegetables before use, if food utensils were clean and stored in a clean area, if vectors or rodents were not seen in the food storage area, if households did not use leftover foods or reheat thoroughly before use, and if a person who is responsible for food preparation washed his/her hands before food preparation or if all family members washed their hands before eating, after visiting the toilet, and after of any manual works.

#### Waste management practices

Households’ sanitation performance was taken as “adequate” if all the family members usually use a sanitary latrine, if functional handwashing facility was available around the latrine, if the living environment is free from rubbish and human and animal excreta, and if households disposed wastes hygienically.

### Data management and statistical analysis

Data were entered using EPI-INFO version 3.5.3 statistical package and export into Statistical Package for Social Sciences (SPSS) version 20 for further analysis. For most variables, data were presented by frequencies and percentages. Univariable binary logistic regression analysis was used to choose sanitation variables for the multivariable binary logistic regression analysis, and variables which had *p* value less than 0.2 by the univariable analysis were then analyzed by multivariable binary logistic regression for controlling the possible effect of confounders like the age of children, maternal education, and paternal education. Finally, variables which had significant association were identified on the basis of AOR with 95% CI and *p* < 0.05.

## Results

### Socio-demographic information of children and mothers

In this study, a total of 225 children participated with 100% response rate. The response rate was high because we collected and analyzed stool by moving from village to village with relevant treatment of infected children. Out of 225 children, 119 (52.9%) were females. One hundred sixty-six (73.8%) of the children were aged between 24 and 59 months. The median age of children was 42 months, and the interquartile range was 24–48 months. One hundred thirty-four (59.6%) mothers aged 30 years and below. The fast majority, 180 (80.0%), of the mothers and nearly two thirds, 139 (65.0%), of the fathers did not attend formal education (Table 1).

**Table 1:** Socio-demographic information of households with children aged 6–59 months in rural Dembiya, northwest Ethiopia, May 2017

Variables	Frequency	Percent
Sex of children		
Male	106	47.1
Female	119	52.9
Age of children		
6–24	59	26.2
> 24	166	73.8
Maternal education		
No formal education	180	80.0
Have formal education	45	20.0
Paternal education (*n* = 214)		
No formal education	139	65.0
Have formal education	75	35.0

### Prevalence of intestinal parasitic infections

**Fig. 1:**
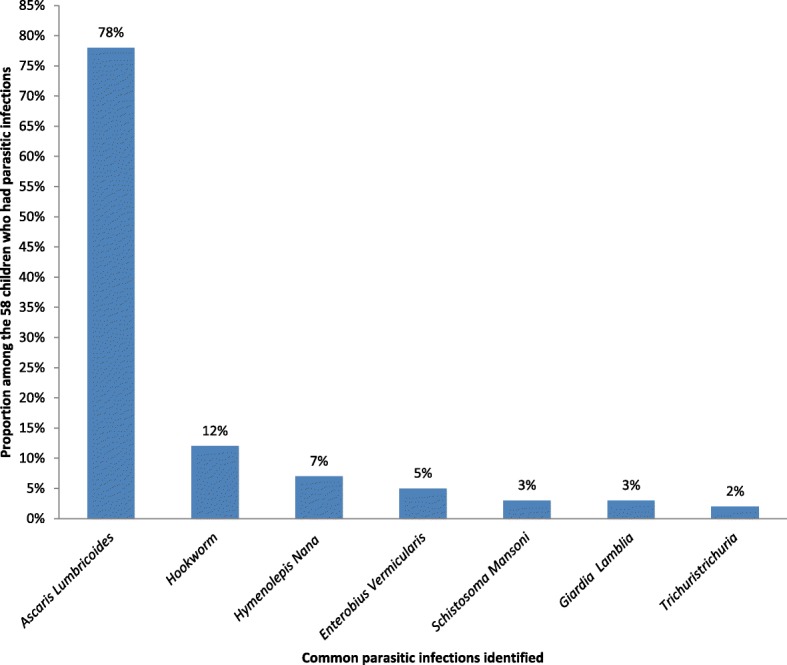
Common intestinal parasitic infections identified among children aged 6–59 months in rural Dembiya, northwest Ethiopia, May 2017

From a total of 225 children investigated, 58 of the children were infected with one or more intestinal parasitic infections. The prevalence of intestinal parasitic infections among children aged 6–59 months in rural Dembiya was therefore found to be 25.8% (95% CI = 20.3–32.0%). The commonest intestinal parasitic infection identified among children was *Ascaris lumbricoides*, which accounted 45 (77.6%) (Fig. 1).

Besides prevalence, the intensity of infection was estimated from the number of eggs per gram (epg) of stool. Accordingly, the intensity of *Ascaris lumbricoides* infection was light (1-4999 epg). Similarly, the intensity of all other intestinal parasitic infections was light (see the classification of intensity of infections in the “Methods” section). The load of the egg of *Ascaris lumbricoide* was ranged from 24 to 5160 epg of stool. Twenty-four to 336 eggs of *Ascaris lumbricoide* per gram of stool were found among 29 (64.3%) of infected children. Nine (19.8%) of the infected children had 360 to 961 eggs of *Ascaris lumbricoide* per gram of stool, and the rest, seven (15.4%) had 1056 to 5160 epg of stool. The egg load of hookworm among the infected children was from 24 to 792 epg of stool. Five of hookworm-infected children had 24 to 48 epg of stool, and the rest, two had 120 and 792 epg of stool. Two of the children infected by *Hymenolepis nana* had 72 epg, and the rest, two had 120 epg of stool. All of the three infected children by *Enterobius vermicularis* had 241 epg of stool, and 48 eggs of *Schistosoma mansoni* per gram of stool was found among the two infected children.

### Hygiene of children

Almost all 222 (98.7%) of the children were unhygienic. Dirt was clearly seen on their fingers, face, hair, body, and clothes. Two thirds, 152 (67.6%), of the households reported that they washed their child’s face with clean water every morning. One hundred fifty-eight (70.2%) of the households reported that their child washed its body once in 3 days. Nearly three fourth, 166 (73.8%), of the children did not keep their fingernail short. One hundred sixty (71.1%) of the children were barefooted at the time of the survey. Below half, 102 (45.3%), of the households said that they frequently washed the hands of their children after playing, defecation, and before eating. Only a quarter, 55 (24.4%), of mothers or caregivers washed their hands properly in different pick times (Table 2).

**Table 2:** Personal hygiene of children aged 6–59 months in rural Dembiya, northwest Ethiopia, May 2017

Hygiene variables	Frequency	Percent
General cleanliness of children		
Clean	3	1.3
Not clean	222	98.7
Children wash their face with clean water in every morning		
Yes	152	67.6
No	73	32.4
Children wash their body with clean water and soap		
Once in 3 days	158	70.2
Once a week	67	29.8
Children’s fingernails kept short		
Yes	59	26.2
No	166	73.8
Children wear shoes		
Yes	65	28.9
No	160	71.1
Children frequently wash their hands after playing, defecation, and before eating		
Yes	102	45.3
No	123	54.7
Mothers’ or caregivers’ hand washing habits		
Before meal	223	99.1
After latrine use	154	68.4
After handling baby’s diaper/feces	138	61.3
After meal	209	92.9
Before feeding a child	154	68.4
Before food preparation	201	89.3
After handling rubbish/ animals	112	49.8
Mothers’ or caregivers hand washing practice		
Good	55	24.4
Not good	170	75.6

### Access to drinking water

**Fig. 2:**
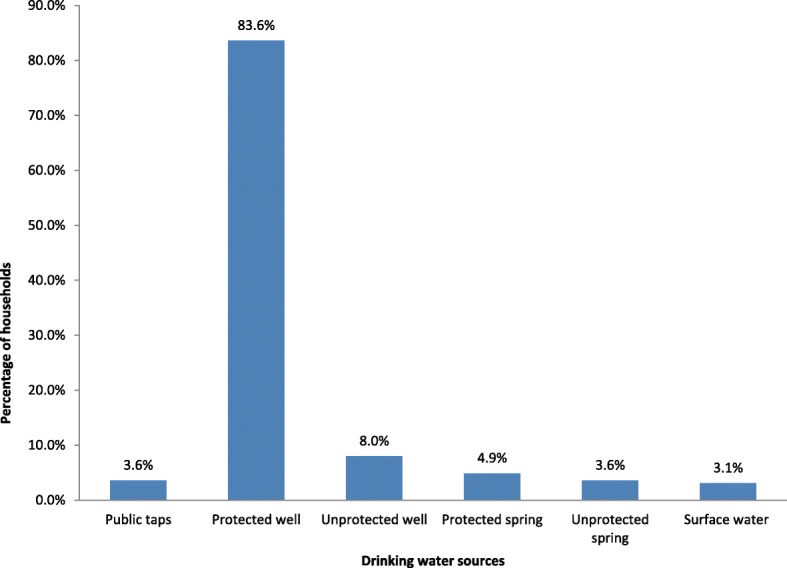
Common drinking water sources for rural households in Dembiya, northwest Ethiopia, May 2017

The majority of households, 190 (84.4%), fetched drinking water from multiple sources. The commonest water sources were protected wells (Fig. 2). The water sources for 166 (73.8%) households were protected. However, the bacteriological analysis of drinking water showed that 158 (70.2%) of the households used water which was not good for consumption, and the water quality of nearly half, 107 (47.6%), of the households was at high-risk level. The great majority, 205 (91.1%), of the households reported that they had access to drinking water throughout the year, and 114 (50.7%) households collected water below 20 l/c/d. The water storage containers in 199 (88.4%) and 147 (65.3%) households were not clean and not properly covered, respectively, at the time of the survey. Home-base water treatment was not commonly practiced in rural Dembiya. Seventeen (7.6%) households treated drinking water at home. One household treated drinking water by solar disinfection. Thirteen households used water guard to treat water at home. Three households boiled drinking water before use (Table 3).

**Table 3:** Quality and access to drinking water in rural Dembiya, northwest Ethiopia, May 2017

Variables	Frequency	Percent
Drinking water quality		
Good	67	29.8
Not good	158	70.2
Risk level of drinking water quality		
Conformity	67	29.8
Low risk	12	5.3
Intermediate risk	39	17.3
High risk	107	47.6
Drinking water sources		
Protected	166	73.8
Unprotected	59	26.2
More than one water source		
Yes	190	84.4
No	35	15.6
The water source discharges water at any time		
Yes	205	91.1
No	20	8.9
Service level of water supply		
Basic access	111	49.3
No access	114	50.7
Cleanliness of drinking water storage containers		
Clean	26	11.6
Not clean	199	88.4
Water storage containers were properly covered at the time of the survey		
Yes	78	34.7
No	147	65.3
Treat water at household level		
Yes	17	7.6
No	208	92.4

### Food safety practices

The food safety practices of 107 (47.6%) households were poor. One hundred forty (62.2%) mothers or caregivers prepared food while they had diarrhea or other communicable diseases. Thirty-one (13.8%) households did not wash fruits and vegetables before preparing for consumption. The majority, 203 (90.2%), of the households did not keep food utensils clean, and food utensils were placed on the floor among 85 (37.8%) households. Very few, 3 (1.3%), households used properly maintained shelves to store food items, and vectors or rodents were observed around food storage areas in 113 (50.2%) households. The overwhelming majority, 204 (90.7%), of the households used leftover foods without reheating. Almost all, 221 (98.2%), households prepared foods in dirty areas (Table 4).

**Table 4:** Food safety practices of rural households in Dembiya, northwest Ethiopia, May 2017

Variables	Frequency	Percent
Food safety practices		
Good	118	52.4
Poor	107	47.6
Prepare food while you have diarrhea/or vomiting or other communicable diseases		
Yes	140	62.2
No	85	37.8
Wash fruits or vegetables before preparing for consumption		
Yes	194	86.2
No	31	13.8
Cleanliness of food utensils		
Clean	22	9.8
Not clean	203	90.2
Where food utensils are stored		
On the floor	85	37.8
In shelves	140	62.2
How prepared foods stored		
Stored at well-designed shelves	3	1.3
Store separate from raw foods	183	81.3
Properly covered	33	14.7
The store is clean, illuminated, and ventilated	6	2.7
Vectors or rodents are seen in food storage area		
Yes	113	50.2
No	112	49.8
Reheat leftover foods to use		
Yes	21	9.3
No	204	90.7
Cleanliness of food preparation area		
Clean	4	1.8
Not clean	221	98.2

### Waste management practices

One hundred twenty-eight (56.9%) of households had access to inadequate sanitation. All the family members did not utilize latrine among 153 (68%) households, and human excreta was observed on the living compound of 101 (44.9%) households. The majority, 201(89.3%), and almost all, 216 (96.0%), households disposed solid and liquid wastes on open field, respectively. Two hundred twelve (94.2%) households did not clean their living compound at a regular basis (Table 5).

**Table 5:** Waste management practices of rural households in Dembiya, northwest Ethiopia, May 2017

Variables	Frequency	Number
Households sanitation performance		
Adequate	97	43.1
Not adequate	128	56.9
All members of the household use latrine		
Yes	72	32.0
No	153	68.0
The living compound is free from human excreta		
Yes	101	44.9
No	124	55.1
Management of solid waste		
Open dump	201	89.3
Sanitary disposal (burning and burying)	24	10.7
Liquid waste management		
Discard into soak pit or absorption pit	9	4.0
Discharge to open field	216	96.0
Do you clean the compound regularly		
Yes	13	5.8
No	212	94.2

### WASH predictors of intestinal parasitic infections

Table 6 shows WASH predictors associated with intestinal parasitic infections among children aged 6–59 months. Intestinal parasitic infections were statistically associated with child hand washing practice, drinking water sources, service level of drinking water supply, food safety practices, and households’ sanitation performance. Socio-demographic confounders like the age of children, maternal education, and paternal education could not pass the model assumptions.

**Table 6:** Factors affecting intestinal parasitic infection among children aged 6–59 months in rural Dembiya, northwest Ethiopia, May 2017

Variables	Parasitic infections		COR with 95% CI	AOR with 95% CI
	Yes	No		
Children wash their hands after playing/defecation and before eating				
Yes	10	92	1	
No	48	75	5.89 (2.79, 12.42)	3.86 (1.53, 9.75)**
Mothers’ or caregivers’ hand washing practice				
Good	10	45	1	
Not good	48	122	1.77 (0.83, 3.79)	2.32 (0.81, 6.68)
Drinking water sources				
Protected	22	144	1	
Unprotected	36	23	10.25 (5.14, 20.41)	7.79 (3.30, 18.40)***
Service level of water supply				
Basic access	24	87	1	
No access	34	80	1.54 (0.84, 2.82)	3.05 (1.28, 7.23)*
Drinking water quality				
Good	13	54	1	
Not good	45	113	1.65 (0.82, 3.32)	1.61(0.64, 4.02)
Food safety practices				
Good	9	109	1	
Poor	49	58	10.23 (4.70, 22.30)	4.33 (1.62, 11.58)**
Households sanitation performance				
Adequate	5	92	1	
Not adequate	53	75	13.00 (4.95, 34.17)	5.01 (1.56, 16.16)*

Hosmer and Lemeshow test = 0.095

*Statistically significant at *p* < 0.05

**Statistically significant at *p* < 0.01

***Statistically significant at *p* < 0.001

Children who did not wash their hands after playing/defecation and before eating had higher odds to have intestinal parasitic infections. The prevalence of intestinal parasitic infections was 3.86 times more likely to be higher among children who did not wash their hands after playing/defecation and before eating [AOR = 3.86, 95% CI = 1.53, 9.75]. The probability of childhood intestinal parasitic infections was 7.79 times more likely to be higher if the households collected drinking water from unprotected sources [AOR = 7.79, 95% CI = 3.30, 18.40]. Intestinal parasitic infections among children were significantly associated with the service level of drinking water supply. Childhood parasitic infections were higher among households who collected water below 20 l/c/d [AOR = 3.05, 95% CI = 1.28, 7.23]. This study revealed that intestinal parasitic infections among children were statistically associated with households’ food safety practices. Childhood intestinal parasitic infections were 4.33 times more likely to be higher among households whose food safety practice was poor [AOR = 4.33, 95% CI = 1.62, 11.58]. Children who live in poor sanitation condition had higher odds to have intestinal parasitic infections compared with their counterparts [AOR = 5.01, 95% CI = 1.56, 16.16].

## Discussion

The prevalence of intestinal parasitic infections reported by this community-based cross-sectional study was 25.8% (95% CI = 20.3–32.0%). This may be due to the fact that the area is characterized by poor hygiene and sanitation conditions. As depicted by this study and reported by the District Health Office, the personal hygiene of children was poor, a greater proportion of caregivers practiced poor hand washing, most of the households accessed poor drinking water, and a significant proportion of households had poor food safety practice and sanitation conditions. Moreover, nearly half of the households had no health and WASH information and that could not practice infection prevention strategies at a regular basis. The prevalence reported by this study was lower than the findings of other similar community-based studies conducted in different parts of Ethiopia, such as Wondo Genet (85.1%) [20] and Hawassa Zuria District (51.3%) [27]. The finding of this study was similar with the findings of other studies in Ethiopia like in Wonji Shoa Sugar Estate (24.3%) [1] and Butajira town (23.3%) [28]. *Ascaris lumbricoides* (78%), hookworm (12%), and *Hymenolepis nana* (7%) were the most common parasitic infections identified in the present study. Even though the prevalence is not the same, other community-based studies also identified these intestinal parasitic infections as common infections [1, 20].

This study revealed that intestinal parasitic infection was higher among children who did not usually wash their hands after playing or defecation and before eating. Other similar studies reported about poor hand washing practice of children and risks of intestinal parasitic infections [29–32]. This can be due to the fact that children catch germs when they touch contaminated objects or surfaces or soil which increases the risk of hand contamination with diseases causing pathogens. Hand washing is the single most effective way to prevent the spread of infections [29, 33–35].

This community-based cross-sectional survey depicted that childhood parasitic infection was statistically associated with unprotected drinking water sources which is congruent with the findings of other studies [36–41]. This can be justified that unprotected sources are prone to contamination with different wastes and pathogenic organisms like cysts of protozoon species and eggs of worms which commonly transmitted to human by ingesting of contaminated water [42–45].

The service level of drinking water supply was significantly associated with intestinal parasitic infections. Childhood parasitic infections were common among households who had no access to water 20 l/c/d and above. This finding is in line with other similar studies [46, 47]. This might be because of the shortage of water to keep personal hygiene. Most of the parasitic infections are fecal-oral [39, 48], meaning cysts of protozoon species and eggs of worms reach to our mouth via contaminated hand. Fecal-oral transmission of diseases is very common among communities characterized by poor hygiene due to the shortage of water [49].

Poor food safety practice was identified as a statistically significant variable with childhood intestinal parasitic infections. This finding is supported by the findings of other studies [37, 50]. The best explanation for this could be that poorly prepared and handled food contains a number of disease-causing pathogenic microorganisms, and these pathogens can enter to our digestive system by ingestion of contaminated food [46, 51–54].

This study showed that intestinal parasitic infections were associated with poor sanitation condition. Poor sanitation (indiscriminate disposal of human excreta, liquid waste, and solid waste) results in disease-causing pathogens, especially infective eggs and larvae of helminths would litter the environment. As these diseases are transmitted by the fecal-oral route or by direct penetration of the skin, the risk of infection will increase with such environmental contamination [9, 13, 16, 51, 52]. Moreover, among human excreta, feces are the most dangerous to health. One gram of fresh feces from an infected person can contain around 10^6^ viral pathogens, 10^6^–10^8^ bacterial pathogens, 10^4^ protozoan cysts, and 10–10^4^ helminth eggs [55].

### Limitation of the study

Though it is highly recommended, this research did not use floatation techniques/McMaster technique to detect hookworm because the McMaster chamber was not available in the country. We used standardized wet mount preparation and Kato-Katz techniques. We examined each specimen within 1 h of sampling time to effectively detect hookworm. Different research articles suggest that intestinal parasitic infection is higher among older children in the 6–59 months age range because older children are more active and contact with fecally contaminated soil while playing, which could predispose them to intestinal parasitic infections [23, 56]. For this reason, the older one was selected in this study for households which had more than one child. Therefore, that fact should be considered when interpreting the estimate of “the prevalence of intestinal parasitic infections among children aged 6–59 months in rural Dembiya”.

## Conclusion

The prevalence of intestinal parasitic infections among children aged 6–59 months in rural Dembiya was high. *Ascaris lumbricoides*, hookworm, *Hymenolepis nana*, *Enterobius vermicularis*, *Schistosoma mansoni*, *Giardia lamblia*, and *Trichuris trichuria* were identified. Child hand washing practice, drinking water sources, service level of drinking water supply, food safety practices, and households’ sanitation performance were the WASH predictors statistically associated with intestinal parasitic infections. Deworming should be done for infected children. Moreover, WASH promotion should be implemented to prevent the occurrence and transmission of intestinal parasitic infections in the long run. The community should have access to facilities like community water supply system, waste management or sanitation facilities, and household-level technologies and materials. Moreover, the community should have access to software services or community behavioral change services to promote WASH.

## Abbreviations

AOR: Adjusted odds ratio
cfu: Colony-forming units
CI: Confidence interval
COR: Crude odds ratio
*E. coli*: *Escherichia coli*
epg: Eggs per gram
JDC: Jewish Distribution Committee
NALA: Neglected Tropical Diseases Advocacy Learning Action
SPSS: Statistical Package for Social Sciences
STH: Soil-transmitted helminth
WASH: Water, sanitation, and hygiene
YLD: Years lived with disability
